# Evaluating Firearm Violence After New Jersey’s Cash Bail Reform

**DOI:** 10.1001/jamanetworkopen.2024.12535

**Published:** 2024-05-22

**Authors:** Jaquelyn L. Jahn, Jessica T. Simes, Jonathan Jay

**Affiliations:** 1 The Ubuntu Center on Racism, Global Movements, and Population Health Equity, Drexel University Dornsife School of Public Health, Philadelphia, Pennsylvania; 2 Department of Sociology, Boston University, Boston, Massachusetts; 3 Department of Community Health Sciences, Boston University School of Public Health, Boston, Massachusetts

## Abstract

**Importance:**

Reducing the pretrial detention population has been a cornerstone of movements to end mass incarceration. Across many US cities, there are ongoing public debates on policies that would end pretrial detention due to the inability to afford bail, with some raising concerns that doing so would increase community violence.

**Objective:**

To evaluate changes in firearm violence after New Jersey’s 2017 bail reform policy that eliminated financial barriers to avoiding pretrial detention.

**Design, Setting, and Participants:**

This case-control study used synthetic control methods to examine changes in firearm mortality and combined fatal and nonfatal shootings in New Jersey (2014-2019). New Jersey was chosen because it was one of the first states to systematically implement cash bail reform. Outcomes in New Jersey were compared with a weighted combination of 36 states that did not implement any kind of reform to pretrial detention during the study period. Data were analyzed from April 2023 to March 2024.

**Exposure:**

Implementation of New Jersey’s cash bail reform law in 2017.

**Main Outcomes and Measures:**

Quarterly rates of fatal and nonfatal firearm assault injuries and firearm self-harm injuries per 100 000 people.

**Results:**

Although New Jersey’s pretrial detention population dramatically decreased under bail reform, the study did not find evidence of increases in overall firearm mortality (average treatment effect on the treated, −0.26 deaths per 100 000) or gun violence (average treatment effect on the treated, −0.24 deaths per 100 000), or within racialized groups during the postpolicy period.

**Conclusions and Relevance:**

Incarceration and gun violence are major public health problems impacting racially and economically marginalized groups. Cash bail reform may be an important tool for reducing pretrial detention and advancing health equity without exacerbating community violence.

## Key Points

**Question:**

Did gun violence in New Jersey increase, decrease, or remain unchanged after implementation of the state’s 2017 bail reform policy?

**Findings:**

In this case-control study, there was no change in fatal and nonfatal gun violence in New Jersey after substantial declines in jail incarceration under bail reform.

**Meaning:**

These findings suggest that bail reform may be an important tool for reducing jail incarceration without exacerbating community gun violence.

## Teaser

This case-control study evaluates changes in firearm violence after New Jersey’s 2017 bail reform policy, which eliminated cash bail in favor of a risk-based approach that uses an empirical risk assessment tool to help determine whether someone is detained pretrial.

## Introduction

Over the past several decades, the incarcerated population in the US has grown by 500%. Approximately 9 million people are incarcerated in jail each year,^1^ about 80% of whom are held pretrial,^2^ meaning they are legally innocent while incarcerated. The inability to afford bail prevents many from being released from jail pretrial, and the average period of jail incarceration is 26 days.^3^ Socioeconomic inequities across racialized groups also make the ability to pay bail, and therefore rates of jail incarceration, highly racially inequitable.^4,5^

Cash bail reform is a major lever for reducing the pretrial jail population, but its implications for community health and well-being remain a sticking point in policy debates. Recently, several counties and states have considered policies that would eliminate financial barriers to bail (ie, end cash bail) and, therefore, greatly reduce jail incarceration.^6^ In 2017, New Jersey implemented one of the most comprehensive bail reforms to date.^7^ The law shifted the state away from a resource-based approach, in which 38% of the state’s jail population was held pretrial because they could not afford bail, to a risk-based approach that uses an empirical risk assessment tool to help determine whether someone is detained pretrial.^8^ New Jersey’s 2017 law virtually eliminated pretrial detention due to the inability to afford bail.^8^ Since the policy was implemented, the number of people detained pretrial has decreased. For example, although 8899 people were held pretrial at the end of 2015, there were 4976 people held pretrial in county jails by the end of 2019.^9^

Few jurisdictions have implemented policies to eliminate cash bail, and bail reform remains an active area of policy debate in New Jersey and across the US. Both arguments for and against bail reform have underscored implications for community safety and well-being, with a particular focus on gun violence.^10^ Importantly, however, nearly all extant studies fail to fully assess these arguments because they have focused solely on criminal justice outcomes for those who were released under the new law, such as rearrest or reincarceration, rather than directly testing whether violence and other health outcomes in the community changed after bail reform.^6,11^

On the one hand, critics of cash bail reform, including in New Jersey, raise concerns that rates of violence would increase in the absence of a broad system of pretrial detention as a tool for incapacitation and crime deterrence.^12,13^ Some scholars have argued that the rise of mass incarceration, even if unjust and inefficient as a crime prevention strategy, likely contributed to reductions in firearm violence from the mid-1990s to the mid-2010s by removing (ie, incapacitating) large shares of the populations (eg, low-income Black male youth) most likely to be involved in firearm violence as victims or shooters.^14^ Other research^11,15^ has evaluated whether the threat of incarceration and criminal punishment deters crime. According to both incapacitation and deterrence theories, reducing the jail population through cash bail reform would tend to increase gun violence in the community, particularly interpersonal violence.

Alternatively, other research suggests rates of violence might decline after bail reform, largely because of reductions in jail incarceration and its widespread harms in the community. Studies of neighborhood collective efficacy,^16,17^ for example, hypothesize that strong social ties are important for gun violence prevention. Under bail reform, there could be fewer disruptions to social support and family caretaking arrangements because fewer community members are in jail.^18,19,20^ Several studies^21,22,23,24,25^ have also shown that psychological and economic stressors are important determinants of violence. Jail incarceration removes people from the labor market, and even for those who are not convicted, the stigma of a criminal record can have negative employment consequences that immediately affect families’ financial well-being.^19^ For children, a family member’s incarceration is an adversity linked with higher rates of fighting at school and suicidal ideation.^26^ Therefore, by keeping social networks intact and preventing some of the adverse consequences of jail incarceration, cash bail reform may reduce rates of violence.

In this study of New Jersey’s bail reform law, we focus on 2 primary community health outcomes: firearm mortality and a combined rate of fatal and nonfatal intentional interpersonal shootings. To evaluate the impact of bail reform on gun violence in New Jersey, we used synthetic control methods (SCM), a robust approach that is preferred for studies with single treated units.^27,28,29^ In addition to overall changes in rates of firearm mortality, we examined changes among Black, White, and Hispanic racialized groups given large inequities in both pretrial detention and gun violence due to structural racism.^30,31^ Currently, the main policy response to gun violence is criminalization and incarceration.^32^ Our study assesses whether cash bail reform was implemented without exacerbating community violence and inequities.

## Methods

This case-control study was approved by the Drexel University institutional review board. Informed consent was not obtained because the data are publicly available and anonymous, in accordance with 45 CFR §46. We followed the Strengthening the Reporting of Observational Studies in Epidemiology (STROBE) reporting guidelines for observational studies.

### Dependent Variables

Our first study outcome is quarterly rates of firearm mortality per 100 000 constructed using deidentified National Center for Health Statistics (NCHS) mortality files from 2014 to 2019, which include the month, year, decedent race and ethnicity, and county of the death occurrence. We limited analyses to firearm-related deaths, which included the following causes of injury: homicide, suicide, unintentional, and undetermined (*International Statistical Classification of Diseases and Related Health Problems, Tenth Revision* codes W32-W34, X72-X74, X93-X95, and Y22-Y24). Our primary analyses examined all types of firearm-related deaths except for suicides, but we secondarily included suicides in a sensitivity analysis. Total counts and rates of all firearm-related deaths before and after the 2017 policy are provided in eTable 1 in Supplement 1.

Our second study outcome is a quarterly rate of fatal and nonfatal shootings per 100 000 using data from the Gun Violence Archive (GVA) from 2014 to 2019. The GVA is a nonprofit organization that uses government, media, and commercial data sources to provide geocoded dataset of firearm violence incidents. The GVA is the most complete firearm violence data source with compiled information for both fatal and nonfatal gun violence during the study period.^33^ The data are publicly available and free to download. GVA data are typically used to measure intentional, interpersonal firearm violence, because intentional self-harm incidents (ie, suicides and suicide attempts) are omitted and unintentional shootings are a small proportion of total incidents. Validation work has found good sensitivity (81%) and excellent specificity (99%) when comparing the GVA with incident-level firearm violence data compiled by local police agencies, which are considered the criterion standard.^34^ We geocoded the GVA location data to US counties and analyzed a combined total count of fatal and nonfatal shootings from 2014 to 2019. For both outcomes (NCHS firearm-related mortality and GVA combined fatal and nonfatal shootings), we estimated a quarterly, county-level rate using the annual county population provided by the US Bureau of the Census County Population Estimates (2014-2019).

### Treated vs Control Units

In our analysis, New Jersey is the single treated unit. To determine which states could contribute to the synthetic control donor pool, we constructed a pretrial detention reform database that documented whether a local or state government implemented pretrial detention reforms during our study period. We examined the 3 years before and after New Jersey implemented its reform on January 1, 2017. The following jurisdictions implemented pretrial cash bail reform during our study period: Alaska; California; Kentucky; New Mexico; Washington, DC; Fulton County, Georgia; Cook County, Illinois; Orleans Parish, Louisiana; Philadelphia County, Pennsylvania; and Harris County, Texas. The following jurisdictions implemented other forms of pretrial detention reform (eg, a risk assessment tool) during our study period: Arizona; Colorado; Connecticut; Hawai’i; Maryland; Missouri; Vermont; Polk County, Iowa; and Davidson County, Tennessee. In addition, Delaware and Rhode Island do not participate in the Census of Jails, a data source for a key covariate, and thus are excluded from the analysis. We excluded these jurisdictions from the potential donor pool by first generating a dataset of county-level covariate conditions (except for state-level corrections expenditures and urbanization), and then aggregating up to 36 control states with excluded counties, states, and Washington, DC.

### Covariates

We chose theoretically motivated covariates that would be associated with pretrial detention policy implementation. To estimate county-level socioeconomic disadvantage, we used the proportion of the county population that is aged 25 years or older with less than a high school degree, the race-income Index of Concentration at the Extremes, a measure of racialized economic segregation,^35^ and county-level labor force participation. We also adjusted for county-level crime and jail incarceration rates and for state-level annual state and local government expenditures on corrections (per capita) and urbanization. Data sources and details on covariate variable construction are available in the eAppendix in Supplement 1.

### Statistical Analysis

Data were analyzed from April 2023 to March 2024. Our analysis used SCMs to compare observed postpolicy trends in gun violence in New Jersey with a strongly matched comparison group comprising 36 jurisdictions that did not implement reforms to pretrial detention during this period. An advantage of SCM over traditional difference-in-differences or controlled interrupted time series methods is that it is less subject to bias from violations of the parallel trends assumption.^36^ Rather than averaging across nontreated areas, we modeled a comparison group on the basis of prepolicy rates of gun violence in New Jersey along with covariates. Augmented SCMs extend traditional SCM to improve the fit of the observed vs synthetic control outcomes in the prepolicy period by allowing donor weights to be negative. This is particularly important in the context of our study because our treated unit had lower prepolicy rates of gun violence overall compared with most control units.^28^ The augmented SCM uses a ridge regression with a regularization parameter that penalizes increasing departure from nonnegative weights,^27^ and our residualized models set the ridge penalty to 0 for covariates but greater than 0 for pretreatment outcomes to generate weights that match on auxiliary covariates.^37^ Covariates were selected from a wide range of theoretically informed county-level and state-level conditions on the basis of the pretrend fit for our synthetic control, and we used root mean squared error to assess goodness of fit. Our final set of covariates included crime and jail incarceration rates, proportion of adults with less than high school education, correction expenditures, urbanicity, and an index of racialized economic segregation.

The average treatment effect on the treated (ATT) is estimated as the difference in the observed outcome vs the weighted synthetic control at each postpolicy time point. The 95% CIs on the ATT were calculated using the conformal inference method.^38^ Separate models were estimated for each study outcome, and NCHS firearm mortality was estimated separately for Black, White, and Hispanic groups. We conducted our analysis using the augsynth package in R statistical software version 4.2.3 (R Project for Statistical Computing). A priori levels of significance were *P* < .05, and all hypothesis tests were 2-sided.

We conducted a series of sensitivity analyses to assess the robustness of our results. First, we repeated our main SCMs but with nonresidualized covariates to see whether our results are consistent across this alternative model specification. Second, we also adjusted for 3 potential confounding factors: state-year gun law restrictiveness (measured as the total number of state firearm law provisions), rates of gun ownership, and state senate majority partisanship. Additional details on these data and sources are available in the eAppendix in Supplement 1. Third, instead of restricting our donor pool to places with no pretrial detention reform policies in general, we broadened our donor pool by excluding only places that implemented bail reform specifically. Fourth, we examined firearm homicide models among male individuals only, to assess whether our findings might be attributable to a law removing firearms from those convicted of domestic violence, which was implemented at the same time as New Jersey’s bail reform. Because intimate partner violence comprises a small proportion of homicide victimization among male individuals (ie, 6%, vs 34% for female individuals),^39^ this specification focused on a population that was unlikely to show substantial benefit from the domestic violence–related firearm restrictions. Finally, we conducted in-time placebo tests, setting the policy implementation year to the first quarters of 2015 and 2016, and in-space placebo tests, setting the implementation state to control states.

## Results

In the 3 years preceding New Jersey’s 2017 bail reform, 1382 people died from firearm injuries (mean [SD], 1.30 [0.05] deaths per 100 000 people annually), and there were 2562 shootings (mean [SD], 2.41 [0.31] shootings per 100 000 people annually). In the 3 years following policy implementation, there were 1219 firearm fatalities (mean [SD], 1.14 [0.13] fatalities per 100 000 people annually) and 2620 shootings (mean [SD], 2.46 [0.16] shootings per 100 000 people annually) (eTable 1 in Supplement 1). Nationally during these years, the mean (SD) annual rate of firearm mortality was higher and increased from 2.79 (0.14) per 100 000 (2014-2016) to 3.01 (0.01) per 100 000 (2017-2019). There were large racialized inequities in firearm mortality in New Jersey that persisted across the study period, with rates among Black people well above the state average during this time (mean [SD], 4.88 [0.52] per 100 000 in the 3 years prepolicy and 3.88 [0.62] per 100 000 in the 3 years postpolicy).

**Figure 1.**
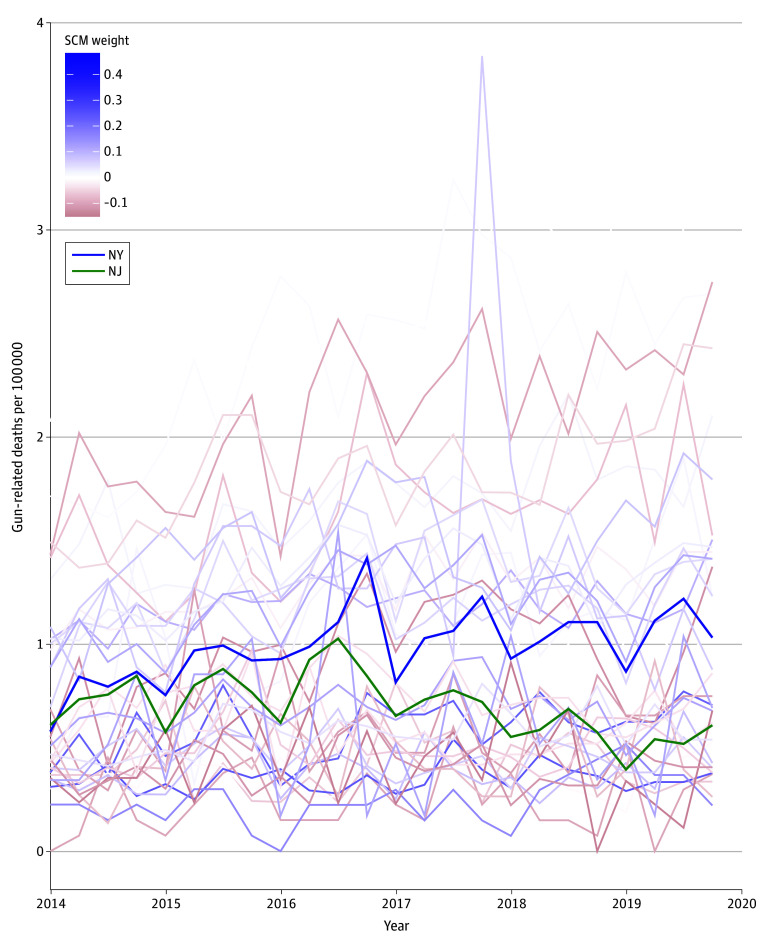
Quarterly State Rates of Firearm Deaths per 100 000 (2014-2017) New Jersey’s (NJ) trend is shown in green. Lines are colored by weights in the augmented synthetic control method (SCM), as shown in the key. NY indicates New York.

**Figure 2.**
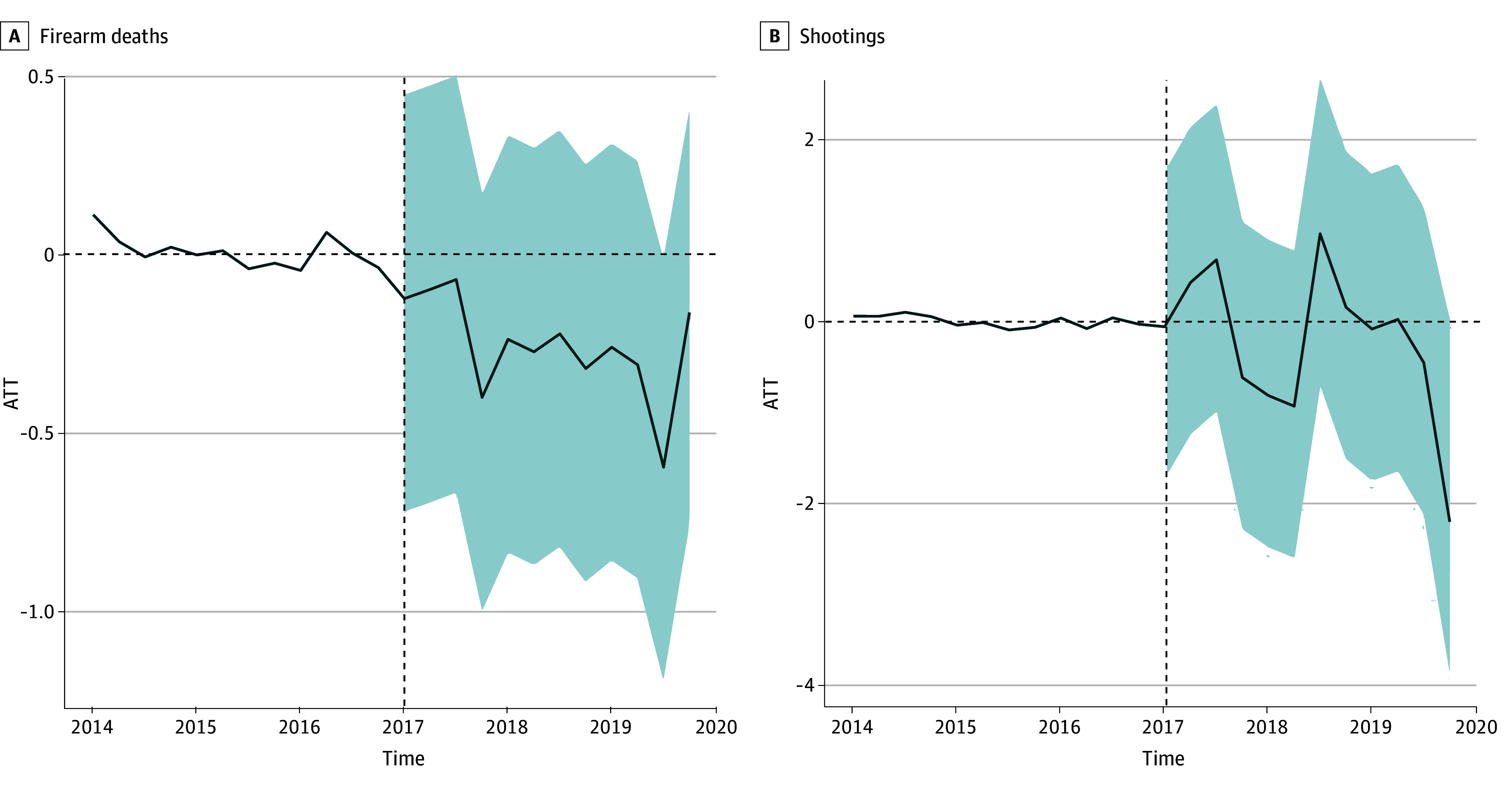
Gap Plots Comparing Rates of Firearm Deaths and Shootings in New Jersey With the Synthetic Control, 2014-2017 Time is measured in quarters since the start of the study period in January 2014. The y-axis shows the difference in the rate of each outcome comparing New Jersey and the synthetic control. The 95% CIs (shaded areas) were calculated using the conformal inference method.^38^ Point estimates and 95% CIs are available in eTable 2 in Supplement 1. ATT indicates average treatment effect on the treated.

We next compared changes in firearm deaths and shootings in New Jersey after bail reform with trends in the synthetic control. For the augmented SCM,^27^ the control group is generated using a weighted combination of states that did not implement bail or any other kind of pretrial detention reform during the study period, and weights can be positive or negative. As shown in Figure 1, the state that contributed most to the synthetic control group and had the strongest positive weight is New York, which closely matched New Jersey’s rates and similarly did not change substantially over the study period. Our gap plots (Figures 2A and 2B) comparing the prepolicy trends in each of our study outcomes suggest a close fit between what was observed in New Jersey and the synthetic control.

Our SCM estimates suggest that there were no significant changes in firearm-related mortality (cumulative ATT, −0.26 deaths per 100 000) or fatal and nonfatal shootings (cumulative ATT, −0.24 deaths per 100 000) in New Jersey after bail reform compared with our SCM. For models estimating NCHS firearm-related mortality, ATT estimates after 2017 ranged from −0.60 (95% CI, −1.20 to 0.00) in quarter 11 to −0.01 (95% CI, −0.71 to 0.46) in quarter 2. For models estimating GVA fatal and nonfatal shootings, ATT estimates after 2017 ranged from −2.20 (95% CI, −3.90 to 0.00) in quarter 12 to 0.97 (95% CI, −0.72 to 2.70) in quarter 7. Figures 2A and 2B plot the ATT, which is the difference in the rate of each outcome comparing New Jersey and the synthetic control. All point estimates for the ATT cluster around 0 (the null), and all 95% CIs include 0 (point estimates and 95% CIs are available in eTable 2 in Supplement 1). Our race-stratified models similarly found no change in firearm-related mortality among Black, White, or Hispanic racialized groups (eTable 2 in Supplement 1). Next, we added suicides to the rate of firearm mortality, which comprised approximately 40% to 50% of firearm deaths over the study period. After adding suicides to the rate of firearm deaths, we found that the synthetic control results were unchanged (eTable 3 in Supplement 1).

We explored the sensitivity of our results to different model specifications, additional covariates, and changing the pool of eligible control states. Our findings were consistent across residualized and nonresidualized models (eTable 3 in Supplement 1). We observed no significant change in firearm deaths or fatal and nonfatal shootings after New Jersey’s bail reform after adjustment for state gun law restrictiveness, gun ownership rates, and state senate partisan control, or when examining firearm mortality among men alone (eTable 3 in Supplement 1). When we broadened our donor pool by only excluding places that implemented bail reform but not other reforms to reduce pretrial detention, results were also null (eTable 3 in Supplement 1). Finally, our in-time placebo test that shifted the policy implementation to the first quarters of 2015 and 2016 found no significant change in firearm-related mortality (eFigure 1 and eFigure 2 in Supplement 1).

## Discussion

In this case-control study, we observed no significant change in firearm mortality or shootings in the 3 years following bail reform in New Jersey, overall and within racialized groups. Our results are consistent with previous evidence that found no increases in new criminal charges against people who were released pretrial under bail reform.^11,40,41,42^ However, previous research has mainly studied outcomes among individuals released pretrial under bail reform, rather than for communities.

Bail reform remains an important tool for reducing pretrial detention and its adverse consequences for individuals and communities. Although we did not find evidence that bail reform was associated with reduced community-level firearm violence (eg, by mitigating the strain of jail incarceration on families and communities), we also did not find evidence supporting incapacitation and deterrence theories that would have suggested that reducing the incarcerated pretrial population by thousands of individuals per year would have increased rates of firearm violence. Supporters of the policy have argued that bail practices effectively criminalize poverty and fuel mass incarceration, with highly racially inequitable consequences.^10^ Thus, bail reform may be warranted on justice and racial equity grounds, even if it does not reduce firearm violence.

There are also important critiques of New Jersey’s risk assessment tool and its implications for increasing racial inequities in pretrial detention, particularly in more recent years that extend beyond our study period.^43^ The risk assessment tool uses 9 risk factors to evaluate the risk of rearrest and failure to appear in court, including factors such as other pending charges or convictions that may reproduce bias in the criminalization of Black and other racially minoritized people. Moreover, there are also charges including murder and serious gun offenses that automatically receive a no-release recommendation, preventing pretrial release and potentially contributing to racial inequities in pretrial detention. New Jersey and other localities are considering revisions and updates to their risk assessment tools, and the impacts of these policy reforms should also be evaluated for their consequences for racial justice and community health equity.^44,45^

Our study contributes to the bail reform policy debate in 3 key ways. First, we move beyond an individualistic model of examining the effects of cash bail reform on recidivism or rearrest by broadening to community-level rates of violence, which speaks to the concerns and issues raised by both proponents and opponents of cash bail reform. Second, we examine multiple measures of firearm violence, drawing from official health statistics and validated crowd-sourced data to study both fatal and nonfatal gun violence. Third, our modeling strategy improves on prior studies by exploiting a novel cash bail intervention at the state level that occurred with enough postintervention observation time to precede the COVID-19 pandemic, which drastically influenced police arrests and jail populations.^46^ Its strengths include comprehensive assessment of gun violence mortality and validated data on shootings, as well as robust covariate adjustment and a strong counterfactual comparison using the synthetic control.

### Limitations

There are also limitations of this study. First, we only analyzed data through 2019 because of the complex changes to court processing, policing practices, poverty, gun violence, and nearly all aspects of social life during the COVID-19 pandemic. Although these factors do not bias our study, we also are not able to provide more updated data on whether the trends we observed in this study persisted after the first 3 years of implementation. Moreover, between 2019 and 2022, the number of people held pretrial in New Jersey has increased.^43^ Second, the generalizability of our results may be limited by the relatively restrictive gun law policy context in New Jersey compared with other US states. Moreover, a law removing firearms from those convicted of domestic violence was also implemented at the same time as New Jersey’s bail reform, and we thus cannot fully distinguish our findings as due to bail reform alone or in combination with this additional policy. In principle, if these domestic violence–related removals reduced overall firearm deaths, they could offset an increase in firearm deaths associated with bail reform. However, a recent review^47^ found limited scientific evidence that this type of firearm restriction reduces overall firearm injury rates, consistent with evidence that the majority of firearm violence is not directed against intimate partners. Moreover, our sensitivity analysis among men alone, for whom intimate partner violence comprises a small proportion of total homicide victimization (6% in 2021, as opposed to 34% for women),^39^ suggests that our main findings are not largely attributable to changes in intimate partner homicide during this period. Third, even before the policy, New Jersey’s jail population was relatively low and decreasing compared with other states, and our findings may not apply to states that have not taken additional steps to reduce jail incarceration.

## Conclusions

Gun violence is a public health crisis in the US, and a main US policy response to gun violence has been increasingly punitive policies. Although the US has staggering levels of gun violence, this study found that community rates of fatal and nonfatal gun violence were not influenced by a policy that reduced levels of pretrial detention. Addressing exposure to firearm violence, and racial inequities therein, requires not only interrupting cycles of violence, but also interrupting cycles of racialized disinvestment and punitive policymaking.

## Notes

Supplement 1. **eAppendix.** Supplemental Methods **eTable 1.** Gun Violence Outcomes 2014-2016 and 2017-2019 in New Jersey, the U.S. on Average, and in the Synthetic Control Donor Pool **eTable 2.** Average Treatment Effect on the Treated and 95% CIs for Each Quarter Post-Policy **eTable 3.** Average Treatment Effect on the Treated and 95% CIs for Each Quarter Post-Policy Robustness Checks **eFigure 1.** Gap Plots for the Actual Policy and Two In-Time Placebos **eFigure 2.** Gap Plots for the Actual Policy and In-Space Placebos

Supplement 2.
Data Sharing Statement
