# Supplementary material for: Evaluating Firearm Violence After New Jersey’s Cash Bail Reform
Source: JAMA Netw Open. 2024 May 22;7(5):e2412535. doi: 10.1001/jamanetworkopen.2024.12535 (PMC11112443; doi:10.1001/jamanetworkopen.2024.12535)
Supplement: Supplement 1. — eAppendix. Supplemental Methods eTable 1. Gun Violence Outcomes 2014-2016 and 2017-2019 in New Jersey, the U.S. on Average, and in the Synthetic Control Donor Pool eTable 2. Average Treatment Effect on the Treated and 95% CIs for Each Quarter Post-Policy eTable 3. Average Treatment Effect on the Treated and 95% CIs for Each Quarter Post-Policy Robustness Checks eFigure 1. Gap Plots for the Actual Policy and Two In-Time Placebos eFigure 2. Gap Plots for the Actual Policy and In-Space Placebos [file jamanetwopen-e2412535-s001.pdf]

Supplemental Online Content

Jahn JL, Simes JT, Jay J. Evaluating firearm violence after New Jersey's cash bail reform. *JAMA Netw Open*. 2024;7(5):e2412535.
doi:10.1001/jamanetworkopen.2024.1253

eAppendix. Supplemental Methods
eTable 1. Gun Violence Outcomes 2014-2016 and 2017-2019 in New Jersey, the U.S. on Average, and in the Synthetic Control Donor Pool
eTable 2. Average Treatment Effect on the Treated and 95% CIs for Each Quarter Post-Policy
eTable 3. Average Treatment Effect on the Treated and 95% CIs for Each Quarter Post-Policy Robustness Checks
eFigure 1. Gap Plots for the Actual Policy and Two In-Time Placebos
eFigure 2. Gap Plots for the Actual Policy and In-Space Placebos

This supplemental material has been provided by the authors to give readers additional information about their work.

© 2024 Jahn JL et al. *JAMA Network Open.*

eAppendix. Supplemental Methods

Data sources

Two covariates are derived from the American Community Survey 5-Year Estimates (2013-2017): the proportion of the county population that is 25 or older with less than a high school degree and the race-income Index of Concentration at the Extremes (ICE). We estimate annual county-level labor force participation using data from the Bureau of Labor Statistics (2014-2019). We calculate a crime rate (combining all reported Part 1 violent and property crimes) from the FBI Uniform Crime Reporting (UCR) Program, which was compiled at the county-level by Jacob Kaplan (2014-2018). Data were not available for 2019, so we use 2018 crime rates for 2019. We estimate the annual average daily jail population the Vera Institute of Justice (2014-2019). Connecticut, Delaware, Hawai'i, Rhode Island, and Vermont do not participate in the Census of Jails, and thus are excluded from the analysis. Hawai'i also implemented pretrial detention reforms during our synthetic control, and thus would be excluded from the donor pool. However, we tried models that remove the jail population rate from the covariates to allow these states to be included in the donor pool, and results were unchanged.

Two state-level covariates are included in the SCM. One covariate is the annual state and local government expenditures on corrections (per capita). These data are derived from the US Bureau of the Census Survey of State and Local Government Finance (2014-2019) and accessed via the Urban-Brookings Tax Policy Center State and Local Finance Data tool. A final covariate is a measure of state-level urbanization. This is calculated as the natural log of the average number of people living within a five-mile radius of a given resident in each census tract in each state and was generated and made publicly available by *FiveThirtyEight*.

In sensitivity analyses reported in eTable 3, we introduce three additional covariates pertaining to the political and policy environment. First, to estimate gun law restrictiveness at the state-year level, we use the annual total number of state firearm law provisions. These data are provided by the State Firearm Law Database (1991-2019) and are publicly available at the Inter-university Consortium for Political and Social Research (ICPSR #37363). Second, gun ownership data are provided by the RAND State-Level Firearm Ownership Database (1980-2016), measured as a latent factor score of estimates of the proportion of adult, noninstitutionalized residents in each state and year who live in a household with a firearm. Because data are not available after 2016, we carry forward estimates in 2016 to subsequent study years (2017-2019). Third, state-year political partisanship was estimated using data gathered by the Correlates of State Policy Project and provided by Shor and McCarty.[1] The measure estimates state-year senate majority party ideological median with a scale ranging from -1 (liberal) to +1 (conservative). Data from this source are not available after 2018, and so for this analysis we carry forward estimates in 2018 to 2019, the final study year.

1. Shor B, McCARTY N. The Ideological Mapping of American Legislatures. *The American Political Science Review*. 2011;105(3):530-551.

© 2024 Jahn JL et al. *JAMA Network Open.*

eTable 1. Gun Violence Outcomes 2014-2016 and 2017-2019 in New Jersey, the U.S. on Average, and in the Synthetic Control Donor Pool

Characteristic	New Jersey		Entire U.S.		SC Donor Pool	
	Pre-Policy	Post-Policy	Pre-Policy	Post-Policy	Pre-Policy	Post-Policy
Firearm Mortality Total	1382	1219	107216	117799	81694	90372
Average Annual Rate of Firearm Mortality	1.30	1.14	2.79	3.01	3.18	3.47
Firearm Suicide Deaths Total	550	567	66400	72311	51315	56313
Average Annual Rate of Firearm Suicide Deaths	0.52	0.53	1.73	1.84	2.17	2.35
Firearm Mortality of Undetermined Intent	[suppressed]	[suppressed]	1445	1432	1189	1109
Average Annual Rate of Undetermined Firearm Mortality	[suppressed]	[suppressed]	0.04	0.04	0.05	0.05
Firearm Homicides	819	643	38511	43019	28516	32135
Average Annual Rate of Firearm Homicides	0.77	0.60	1.00	1.10	0.92	1.03
Firearm Mortality of White People	552	536	68298	72924	53361	57465
Average Annual Rate of Firearm Mortality of White People	0.92	0.89	2.89	3.08	3.23	3.49
Firearm Mortality of Black People	666	529	26180	30009	20790	24134
Annual Average Rate of Firearm Mortality of Black People	4.88	3.88	5.53	6.34	5.50	6.58
Firearm Mortality of American Indian & Alaska Native People	[suppressed]	[suppressed]	1000	1136	643	683

© 2024 Jahn JL et al. *JAMA Network Open.*

Annual Average Rate of Firearm Mortality of American Indian & Alaska Native People	[suppressed]	[suppressed]	3.97	4.51	3.45	3.70
Firearm Mortality of Asian & Pacific Islander People	19	14	1487	1813	836	1090
Annual Average Rate of Firearm Mortality of Asian & Pacific Islander People	0.19	0.14	0.71	0.86	1.08	1.53
Firearm Mortality of Hispanic People	143	140	9904	11639	5786	6810
Annual Average Rate of Firearm Mortality of Hispanic People	0.68	0.66	1.46	1.72	1.55	1.94
Fatal and Non-Fatal Shootings	2562	2620	118847	133029	92094	102521
Annual Average Rate of Fatal and Non-Fatal Shootings	2.41	2.46	3.09	3.39	3.04	3.37
Non-Fatal Shootings	1741	1915	78621	87850	61981	68754
Average Rate of Non-Fatal Shootings	1.64	1.80	2.04	2.24	2.02	2.23

*Rates calculated as per 100,000 population
Counts and annual average rates of firearm mortality were calculated using data from National Center for Health Statistics (NCHS) individual-level mortality files from 2014 to 2019. Counts and annual average rates of fatal and non-fatal shootings were calculated using data from the Gun Violence Archive (GVA) from 2014 to 2019.

© 2024 Jahn JL et al. *JAMA Network Open.*

eTable 2. Average Treatment Effect on the Treated and 95% CIs for Each Quarter Post-Policy

Quarters After 2017	Gun-Related Deaths Overall*		Fatal and Non-Fatal Shootings Overall		Gun-Related Deaths Among Black People*		Gun-Related Deaths Among White People*		Gun-Related Deaths Among Hispanic People*	
	ATT (95% CI)	P-value	ATT (95% CI)	P-value	ATT (95% CI)	P-value	ATT (95% CI)	P-value	ATT (95% CI)	P-value
1	-0.12 (-0.71, 0.46)	0.46	-0.055 (-1.7, 1.6)	0.92	-1.3 (-4.1, 1.5)	0.31	-0.21 (-0.61, 0.18)	0.15	-0.39 (-1.1, 0.27)	0.31
2	-0.099 (-0.68, 0.49)	0.23	0.43 (-1.3, 2.1)	0.31	-0.47 (-3.3, 2.3)	0.15	-0.066 (-0.46, 0.33)	0.31	-0.18 (-0.84, 0.49)	0.15
3	-0.071 (-0.66, 0.51)	0.54	0.68 (-1, 2.4)	0.77	0.13 (-2.7, 2.9)	0.69	-0.031 (-0.42, 0.36)	0.69	-0.27 (-0.93, 0.4)	0.23
4	-0.4 (-0.99, 0.18)	0.38	-0.61 (-2.3, 1.1)	1	-0.93 (-3.7, 1.9)	0.15	-0.53 (-0.92, 0)	0.23	-0.5 (-1.2, 0.17)	0.077
5	-0.24 (-0.82, 0.35)	0.31	-0.81 (-2.5, 0.88)	0.23	-1.6 (-4.4, 1.2)	0.15	-0.095 (-0.49, 0.3)	0.85	-0.38 (-1.1, 0.28)	0.077
6	-0.27 (-0.86, 0.31)	0.15	-0.93 (-2.6, 0.76)	0.31	-2.1 (-4.9, 0.7)	0.15	-0.0097 (-0.4, 0.38)	0.77	-0.36 (-1, 0.31)	0.15
7	-0.22 (-0.81, 0.36)	0.23	0.97 (-0.72, 2.7)	0.23	2 (-0.86, 4.8)	0.54	-0.11 (-0.5, 0.28)	0.54	0.27 (-0.4, 0.94)	0.62
8	-0.32 (-0.9, 0.26)	0.15	0.16 (-1.5, 1.8)	0.92	-1.5 (-4.3, 1.3)	0.15	-0.17 (-0.56, 0.23)	0.31	-0.13 (-0.8, 0.54)	0.38
9	-0.26 (-0.85, 0.32)	0.15	-0.08 (-1.8, 1.6)	0.92	-0.93 (-3.7, 1.9)	0.15	-0.031 (-0.42, 0.36)	0.46	0.088 (-0.58, 0.76)	0.46
10	-0.31 (-0.89, 0.27)	0.15	0.025 (-1.7, 1.7)	1	-0.26 (-3.1, 2.5)	0.54	-0.16 (-0.55, 0.23)	0.31	0.072 (-0.6, 0.74)	0.46
11	-0.6 (-1.2, 0)	0.077	-0.45 (-2.1, 1.2)	0.77	-2.6 (-5.4, 0.19)	0.31	-0.22 (-0.62, 0.17)	0.15	0.22 (-0.45, 0.88)	0.54
12	-0.16 (-0.75, 0.42)	0.23	-2.2 (-3.9, 0)	0.077	-0.11 (-2.9, 2.7)	0.77	-0.028 (-0.42, 0.36)	0.38	0.64 (-0.025, 1.3)	0.46

*Outcome excludes suicides
The ATT is the difference in the rate of each outcome comparing New Jersey and the synthetic control

© 2024 Jahn JL et al. *JAMA Network Open.*

eTable 3. Average Treatment Effect on the Treated and 95% CIs for Each Quarter Post-Policy Robustness Checks

Quarters After 2017	Gun-Related Deaths Overall (non-residualized ASCM model)*		Fatal and Non-Fatal Shootings Overall (non-residualized ASCM model)		Gun-Related Deaths Adjusting for Gun Ownership & Gun Law Restrictiveness*		Gun-Related Deaths Adjusting for Political Controls*		Gun-Related Deaths, All cash bail-related policies removed from the donor pool*	
	ATT (95% CI)	P-value	ATT (95% CI)	P-value	ATT (95% CI)	P-value	ATT (95% CI)	P-value	ATT (95% CI)	P-value
1	-0.098 (-0.55, 0.35)	0.38	0.12 (-0.6, 0.85)	1	0.14 (-0.43, 0.71)	0.38	-0.0087 (-0.61, 0.59)	0.92	-0.14 (-0.87, 0.58)	0.077
2	-0.075 (-0.52, 0.38)	0.46	-0.024 (-0.75, 0.7)	1	0.0047 (-0.56, 0.57)	1	-0.095 (-0.7, 0.51)	0.62	-0.13 (-0.86, 0.59)	0.077
3	-0.045 (-0.5, 0.41)	0.69	0.047 (-0.68, 0.77)	1	-0.13 (-0.7, 0.44)	0.46	-0.09 (-0.69, 0.51)	0.85	-0.073 (-0.8, 0.65)	0.46
4	-0.5 (-0.96, 0)	0.23	-0.3 (-1, 0.42)	1	-0.38 (-0.95, 0.18)	0.077	-0.5 (-1.1, 0.099)	0.15	-0.48 (-1.2, 0.24)	0.077
5	-0.26 (-0.71, 0.19)	0.15	-0.66 (-1.4, 0.066)	0.46	-0.32 (-0.89, 0.25)	0.077	-0.27 (-0.88, 0.33)	0.23	-0.45 (-1.2, 0.28)	0.38
6	-0.24 (-0.7, 0.21)	0.15	-0.043 (-0.77, 0.68)	1	-0.25 (-0.82, 0.32)	0.15	-0.22 (-0.82, 0.39)	0.15	-0.39 (-1.1, 0.34)	0.077
7	-0.12 (-0.57, 0.33)	0.31	0.13 (-0.59, 0.86)	1	-0.27 (-0.84, 0.3)	0.15	-0.27 (-0.88, 0.33)	0.15	-0.36 (-1.1, 0.36)	0.077
8	-0.17 (-0.62, 0.28)	0.23	-0.21 (-0.93, 0.52)	0.77	-0.24 (-0.81, 0.33)	0.15	-0.3 (-0.9, 0.3)	0.15	-0.4 (-1.1, 0.33)	0.077
9	-0.2 (-0.65, 0.25)	0.15	-0.037 (-0.76, 0.69)	1	-0.24 (-0.81, 0.33)	0.15	-0.22 (-0.82, 0.38)	0.23	-0.35 (-1.1, 0.37)	0.23
10	-0.11 (-0.56, 0.34)	0.46	0.46 (-0.26, 1.2)	0.54	-0.27 (-0.84, 0.3)	0.23	-0.32 (-0.92, 0.28)	0.15	-0.45 (-1.2, 0.27)	0.23
11	-0.33 (-0.78, 0.12)	0.077	-0.47 (-1.2, 0.26)	0.54	-0.57 (-1.1, 0)	0.077	-0.6 (-1.2, 0.0051)	0.077	-0.54 (-1.3, 0.18)	0.077
12	-0.1 (-0.55, 0.35)	0.46	-0.73 (-1.5, 0)	0.46	-0.18 (-0.75, 0.39)	0.23	-0.15 (-0.75, 0.45)	0.54	-0.17 (-0.89, 0.55)	0.077

*Outcome excludes suicides
The non-residualized ASCM model does not set the ridge penalty to zero for covariates.

© 2024 Jahn JL et al. *JAMA Network Open.*

eTable 3. Cont.

Quarters After 2017	Gun-Related Deaths Including Suicides		Firearm Homicides, Among Men Only		Multi-outcome ASCM: Gun-Related Deaths Overall*		Multi-outcome ASCM: Fatal and Non-Fatal Shootings Overall	
	ATT (95% CI)	P-value	ATT (95% CI)	P-value	ATT (95% CI)	P-value	ATT (95% CI)	P-value
1	0.14 (-0.44, 0.71)	0.77	-0.099 (-0.58, 0.38)	0.23	0.091 (-0.14, 0)	0.61	-0.14 (-1.3, 0)	0.61
2	-0.0067 (-0.58, 0.57)	1	-0.12 (-0.59, 0.36)	0.15	0.16 (-0.073, 0)	0.38	-0.49 (-1.7, 0)	0.38
3	-0.12 (-0.7, 0.45)	0.92	0.018 (-0.46, 0.49)	0.85	0.12 (-0.11, 0)	0.23	0.27 (-0.9, 0)	0.23
4	-0.48 (-1.1, 0.091)	0.31	-0.32 (-0.79, 0.16)	0.077	-0.15 (-0.38, 0)	0.6	-1.4 (-2.5, 0)	0.6
5	-0.34 (-0.92, 0.23)	0.85	-0.092 (-0.57, 0.38)	0.31	-0.016 (-0.25, 0)	0.16	-0.4 (-1.6, 0)	0.16
6	-0.39 (-0.97, 0.18)	0.15	-0.22 (-0.7, 0.25)	0.15	-0.096 (-0.33, 0)	0.31	0.094 (-1.1, 0)	0.31
7	-0.37 (-0.95, 0.2)	0.15	-0.15 (-0.63, 0.33)	0.15	-0.12 (-0.35, 0)	0.16	-0.26 (-1.4, 0)	0.16
8	-0.098 (-0.67, 0.48)	0.54	-0.35 (-0.83, 0.12)	0.077	-0.11 (-0.34, 0)	0.39	-0.44 (-1.6, 0)	0.39
9	-0.32 (-0.89, 0.26)	0.15	-0.23 (-0.71, 0.25)	0.15	-0.032 (-0.26, 0)	0.33	0.13 (-1, 0)	0.33
10	-0.2 (-0.78, 0.37)	0.23	-0.29 (-0.77, 0.18)	0.15	-0.053 (-0.28, 0)	0.078	0.37 (-0.8, 0)	0.078
11	-0.33 (-0.9, 0.25)	0.15	-0.44 (-0.92, 0.032)	0.077	-0.22 (-0.45, 0)	0.15	-0.94 (-2.1, 0)	0.15
12	-0.24 (-0.81, 0.34)	0.31	-0.13 (-0.6, 0.35)	0.15	-0.02 (-0.25, 0)	0.3	-0.69 (-1.9, 0)	0.3

© 2024 Jahn JL et al. *JAMA Network Open.*

eFigure 1. Gap Plots for the Actual Policy and Two In-Time Placebos

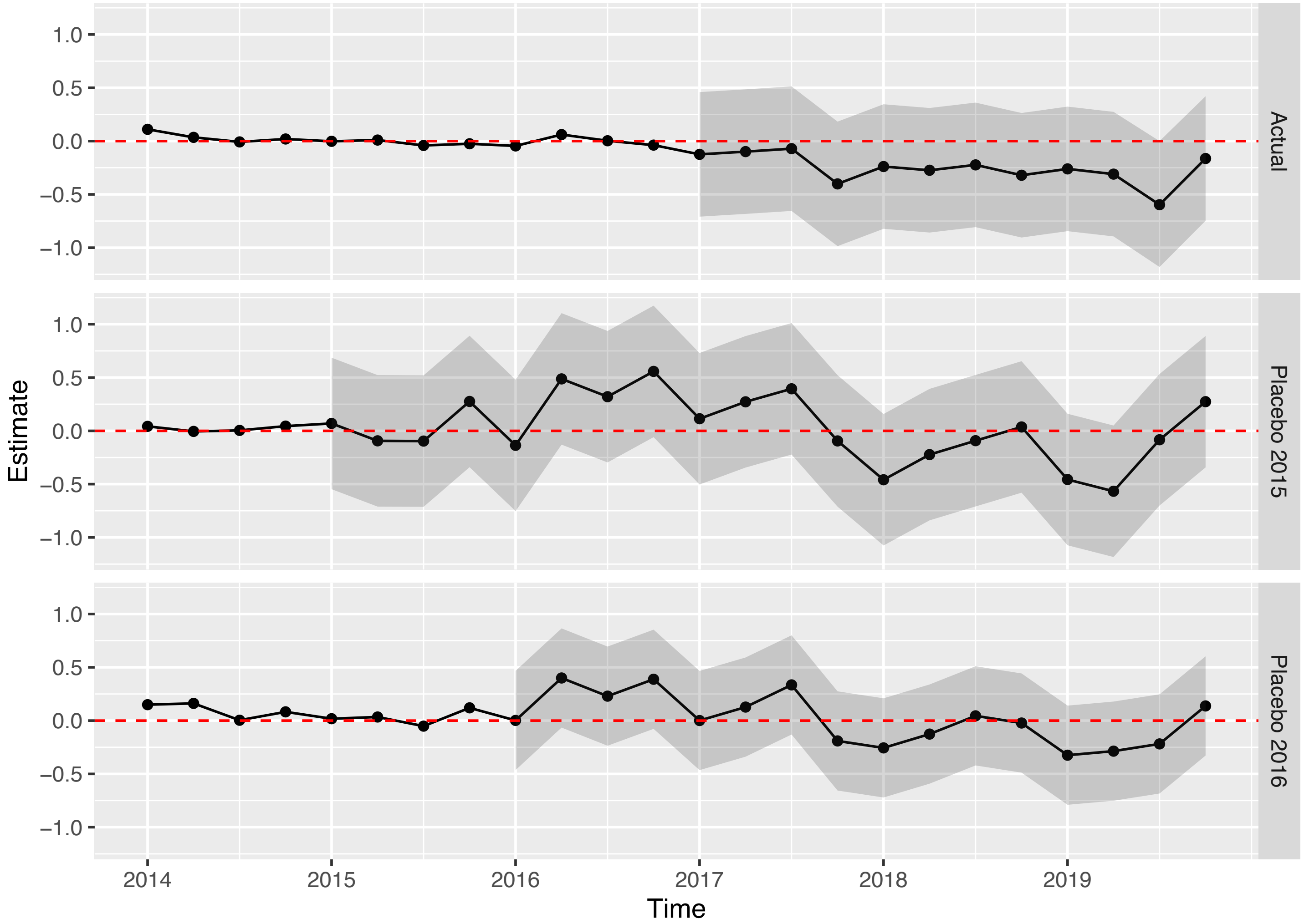

In-time placebo tests set the policy implementation year to be the first quarters of 2105 and 2016, compared with the actual policy implementation at the start of 2017. The outcome for these models was quarterly rates of firearm mortality excluding suicides from NCHS.

© 2024 Jahn JL et al. *JAMA Network Open.*

eFigure 2. Gap Plots for the Actual Policy and In-Space Placebos

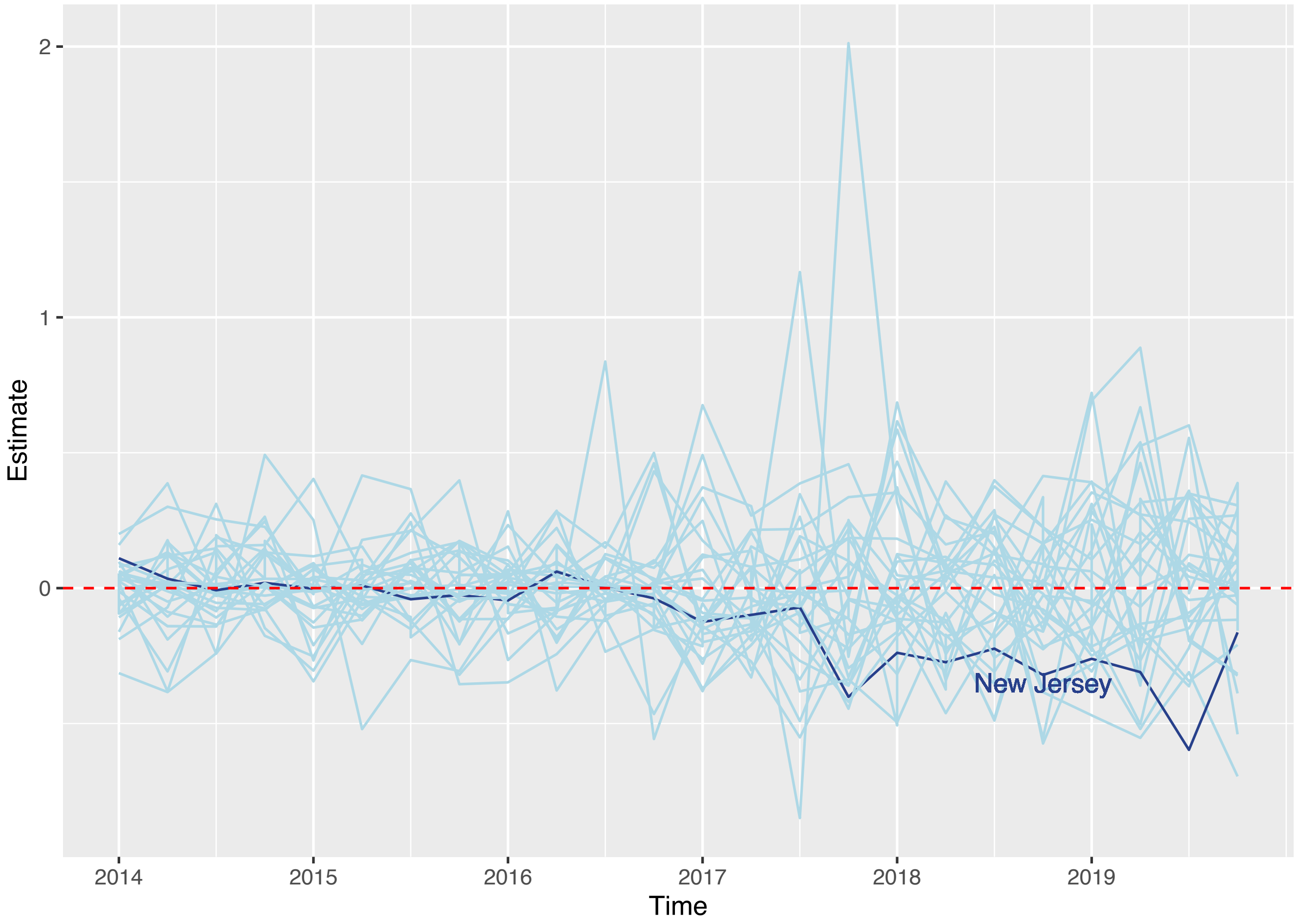

In-space placebo tests set the policy implementation to have occurred in each of the control states, as compared with the actual policy implementation which occurred in New Jersey. The outcome for these models was quarterly rates of firearm mortality excluding suicides from NCHS.

© 2024 Jahn JL et al. *JAMA Network Open.*
