# Supplementary material for: Evaluating Firearm Violence After New Jersey’s Cash Bail Reform
Source: JAMA Netw Open. 2024 May 22;7(5):e2412535. doi: 10.1001/jamanetworkopen.2024.12535 (PMC11112443; doi:10.1001/jamanetworkopen.2024.12535)
Supplement: Supplement 2. — Data Sharing Statement [file jamanetwopen-e2412535-s002.pdf]

Data Sharing Statement

Jahn. Evaluating Firearm Violence After New Jersey's Cash Bail Reform. *JAMA Netw Open*. Published May 22, 2024. doi:10.1001/jamanetworkopen.2024.12535

Data
Data available: Yes
Data types: Deidentified participant data
How to access data: Upon publication, GVA data will be made publicly available on Harvard Dataverse. NCHS mortality data must be accessed through NCHS.
When available: With publication

Supporting Documents
Document types: Statistical/analytic code
How to access documents: Upon publication code will be made publicly available on Harvard Dataverse
When available: With publication

Additional Information
Who can access the data: Data will be made publicly available
Types of analyses: Data will be made available for replication purposes.
Mechanisms of data availability: Data will be made publicly available
